# The Physiological Role of Ghrelin in the Regulation of Energy and Glucose Homeostasis

**DOI:** 10.7759/cureus.7941

**Published:** 2020-05-03

**Authors:** Alina Sovetkina, Rans Nadir, Jerry Ngai Man Fung, Ashkan Nadjarpour, Benjamin Beddoe

**Affiliations:** 1 Faculty of Medicine, Imperial College London, London, GBR; 2 Medicine, Imperial College London, London, GBR

**Keywords:** ghrelin hormone, glucose, nutrition and metabolism, obesity, homeostasis, diabetes

## Abstract

Ghrelin is a peptide hormone that is primarily released from the stomach. It is best known for its role in appetite initiation. However, evidence also suggests that ghrelin may play a much wider role in energy homeostasis and glucose metabolism. It is known that exogenous ghrelin exerts an orexigenic signal via growth hormone secretagogue receptors in the arcuate nucleus of the hypothalamus. However, blocking ghrelin signalling in the arcuate nucleus does not decrease feeding. Evidence now proposes that an alternative pathway for ghrelin’s action is via the vagus nerve. Furthermore, it has been suggested that ghrelin signalling is an important physiological regulator of body adiposity and energy storage. Ghrelin also seems to be important in controlling glucose metabolism through action in the pancreatic islets of Langerhans, representing a promising novel therapeutic target in diabetes treatment. Despite these findings, further research in humans is required before ghrelin can be indicated as a therapeutic target in obesity or diabetes. This review summarises the current literature concerning ghrelin’s physiological roles in energy and glucose homeostasis.

## Introduction and background

Energy homeostasis comprises a balance of energy intake and energy expenditure, influenced by both energy storage and food intake [1]. Glucose homeostasis involves maintaining blood glucose levels through a balance of insulin and glucagon [2]. Ghrelin is an orexigenic hormone implicated in the physiological regulation of both processes, through its activation of the growth hormone secretagogue receptor 1a (GHSR) [3]. The ghrelin gene encodes pre-proghrelin, which produces different subproducts; acylated active form of ghrelin and des-acyl-ghrelin. Acylation is mediated by the enzyme ghrelin-O-acyl transferase (GOAT) [4]. This review will discuss acylated ghrelin.

Ghrelin regulates both short-term energy balance, through appetite control, and long-term, through changes in body weight [4]. Ghrelin also regulates blood glucose through the control of insulin and glucagon secretion [5]. This review will explore ghrelin’s physiological role in multiple facets of energy and glucose metabolism. In particular, central pathways mediating ghrelin’s physiological role in appetite control will be discussed, as well as ghrelin’s effects on adipose tissue and blood glucose levels.

## Review

Ghrelin’s effect on appetite and weight gain

Food intake is an important aspect of both energy and glucose homeostasis [1]. Ghrelin’s main role is suggested to be appetite stimulation, and thus energy intake. Ghrelin secretion is stimulated in negative energy balance states during fasting, and suppressed during positive energy balance states post-feeding, suggesting ghrelin has a compensatory role on energy imbalance [6].

Pharmacological evidence supports ghrelin’s role in appetite stimulation. Chronic intracerebroventricular (ICV) ghrelin infusion strongly increases feeding and body weight gain in rodents, whilst administration of anti-ghrelin immunoglobulin suppresses feeding [7]. Ghrelin levels are shown to closely follow feeding schedules. Plasma ghrelin increases by approximately two-fold before a meal and falls shortly after in humans [8]. This suggests ghrelin plays a role in feeding initiation. However, set meal times meant the subjects were aware of when food was to be given. Therefore, meal initiation may not have been influenced by circulating ghrelin levels, but by an anticipatory response to the food.

Conversely, physiological studies question the necessity of ghrelin in appetite and weight regulation. Evidence shows indistinguishable food intake and body weight difference between ghrelin-knockout (KO) and control mice on administration of either high-fat diet (HFD) or standard chow diet [9,10]. However, such results in germline ghrelin KO-models could be explained by developmental compensation as a result of lifelong ghrelin deficiency. Nevertheless, a receptor KO study supports ghrelin’s importance in appetite and energy storage regulation. Zigman et al. demonstrated both male and female GHSR-KO mice had lower food intake and body weight gain on HFD compared to control mice. Lower body weight of GHSR-KO mice on HFD suggests ghrelin causes body weight change in over-fed states, adapting to a positive energy balance by increasing fat storage [11]. As can be seen, KO-models are inconclusive as to ghrelin’s influence on appetite and weight gain. Study differences between KO-models may be explained by slight variations in mice genetic backgrounds, which may influence feeding and body weight [12]. Ablation models that bypass neonatal compensation may be better suited to evaluating ghrelin’s physiological role. Diphtheria-toxin mediated ablation of ghrelin-expressing cells in adult mice had no effect on feeding or body weight, either short or long-term [13]. This also suggests ghrelin does not play a physiological role in appetite or weight control. However, in this study, plasma ghrelin only declined to 80-95%. The remaining ghrelin may have been enough to maintain effects on feeding and body weight.

Overall, most physiological evidence suggests ghrelin does not regulate food intake and body weight. However, pharmacological and some physiological evidence does suggest ghrelin regulates such endpoints, at least under HFD, and thus is involved in energy homeostasis. Ghrelin’s more significant role is suggested to be in glucose homeostasis regulation, especially under negative energy balance which is explored below.

Central ghrelin action

Ghrelin’s orexigenic role is suggested to be centrally mediated [4]. C-fos expression-mapping following ICV ghrelin administration showed fos-immunoreactive neurones in regions primarily implicated in appetite control. This included the arcuate nucleus (ARC) and the ventromedial-nucleus of the hypothalamus (VMH) [7]. This distribution followed that of GHSR [14]. Furthermore, double immunohistochemistry revealed ghrelin-induced c-fos expression in Neuropeptide Y (NPY) neurones also [7]. It is well-documented that NPY neurones within the ARC co-express agouti-related protein (AgRP) [15].

Selective ablation of AgRP neurones with diphtheria toxin in adult mice prevented orexigenic response to exogenous ghrelin [16]. NPY/AgRP double-knockout mice failed to increase feeding in response to ghrelin administration [17]. This shows NPY/AgRP neurones are necessarily for ghrelin’s orexigenic action. However, these studies still rely on exogenous ghrelin rather than assessing the physiological role of ghrelin. Selective GHSR-knockouts from specifically NPY/AgRP neurones would elucidate whether ghrelin plays a physiological role through NPY/AgRP neurones. Moreover, this evidence is in rodent models which may not be representative of mechanisms in other species. Although ghrelin has been demonstrated to exert orexigenic effects through NPY/AgRP neurones, ghrelin-KO models suggest the presence of ghrelin is not essential for NPY/AgRP control of appetite. Northern blot analysis revealed no significant difference in AgRP or NPY expression between control and ghrelin-KO mice [9]. However, although NPY and AgRP protein expression was unaffected, physiological levels of ghrelin may still have an effect on NPY/AgRP neuronal firing. Indeed, ghrelin increases c-Fos expression in the ARC of mice with selective knock-in of GHSR in AgRP neurones [18]. However, this study again uses exogenous ghrelin which may overstate its physiological role in stimulating AgRP neurones.

Ghrelin’s action on the central nervous system (CNS), specifically AgRP neurones, has more solidly been linked to glucose homeostasis. AgRP neurone-selective GHSR re-expression in GHSR-KO calorie-restricted mice normalised blood glucose and glucagon levels [18]. This demonstrates the importance of ghrelin’s glucoregulatory action within the ARC under conditions of calorie restriction.

**Figure 1:**
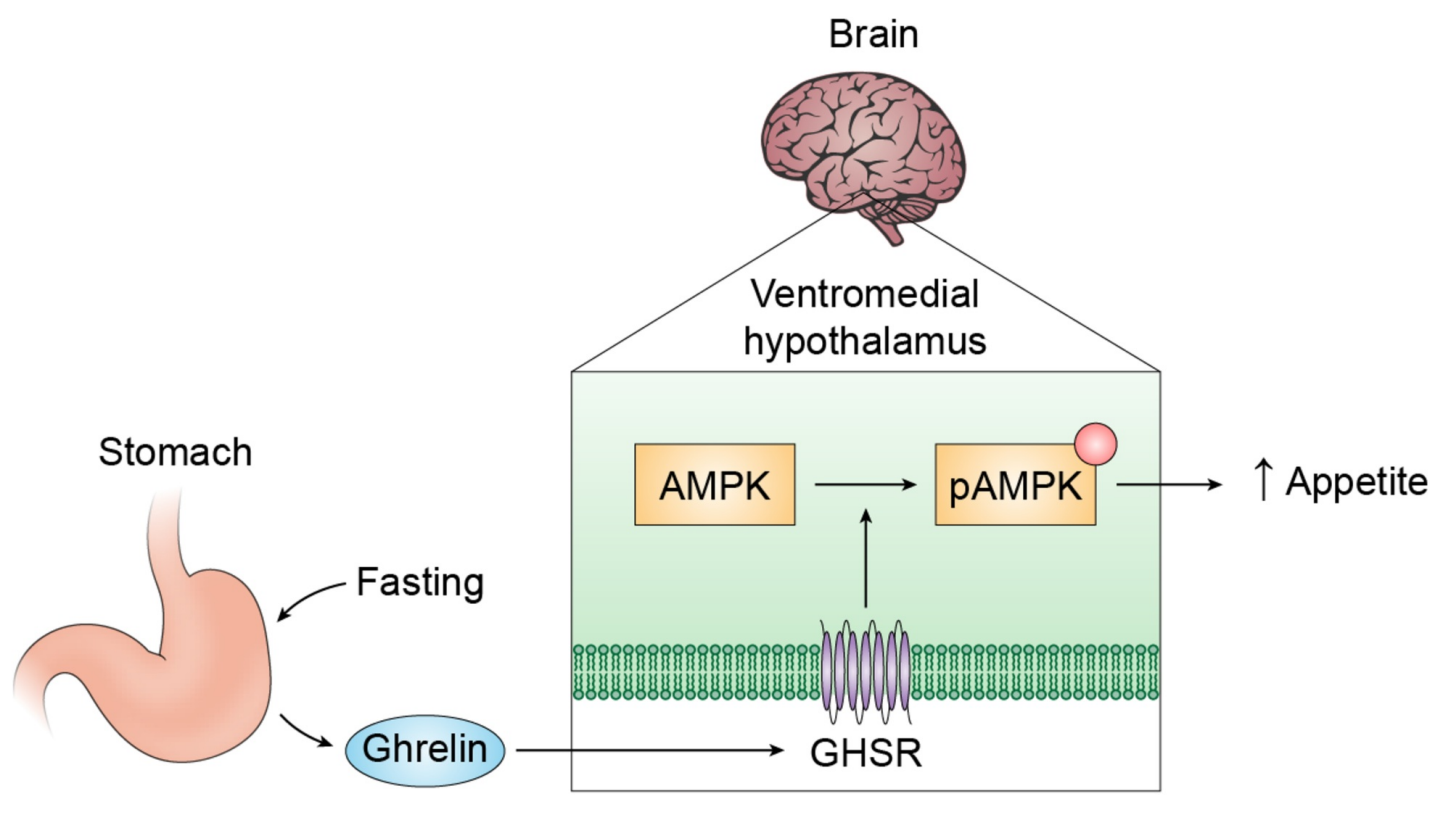
A simplified proposed model for ghrelin’s orexigenic action through AMP-AMPK in the VMH Fasting stimulates ghrelin production by endocrine cells of the stomach [21]. Ghrelin acts on GHSR on the VMH to phosphorylate AMPK. This results in a change in fatty acid biosynthesis (not shown), resulting in increased feeding. GHSR: Growth hormone secretagogue receptor; VMH: Ventromedial nucleus of the hypothalamus; AMPK: 5' adenosine monophosphate-activated protein kinase; pAMPK: phosphorylated 5' adenosine monophosphate-activated protein kinase.

Evidence suggests ghrelin regulates energy balance through 5' adenosine monophosphate-activated protein kinase (AMPK) in the hypothalamus (see Figure 1). AMPK potentially regulates energy balance through appetite stimulation [19]. Increased ghrelin levels during fasting have been suggested to stimulate feeding via AMPK-mediated suppression of certain enzymatic steps in fatty acid biosynthesis in the VMH [20]. Fatty-acid-synthase (FAS) is a key enzyme in fatty acid biosynthesis. Fasting selectively decreases FAS messenger ribonucleic acid (mRNA) expression in the VMH. This was shown to be dependent on ghrelin, as VMH FAS mRNA did not decrease in GHSR-KO mice when fasted. Central administration of an AMPK-inhibitor to ghrelin-treated rats blocked ghrelin-induced decrease of FAS mRNA expression in the VMH [20]. This suggests ghrelin’s inhibition of FAS is mediated through AMPK. This provides evidence that ghrelin exerts its orexigenic effect through activation of AMPK in the VMH.

To summarise, evidence suggests ghrelin exerts its orexigenic actions through NPY/AgRP neurones in the ARC. However, the use of exogenous ghrelin limits these studies. More physiological studies are required to confirm the necessity of NPY/AgRP neuronal signalling in ghrelin’s role in homeostasis. More solid evidence exists demonstrating the ARC is important in ghrelin’s role in glucose metabolism.

Vagus nerve and ghrelin

Vagal neurons have been proposed as an alternative pathway for ghrelin’s orexigenic actions. The afferent vagus is thought to convey appetite-regulating signals from the gastrointestinal tract to the hypothalamus via the nucleus tractus solitaris in the brainstem, resulting in adaptive changes in energy homeostasis [22]. GHSR found in the dorsal vagal complex suggest it is a target for circulating ghrelin [23]. Williams et al. examined the role of the vagus nerve in plasma ghrelin regulation during fasting by carrying out subdiaphragmatic vagotomy in rats. Whilst vagotomy did not suppress baseline ghrelin levels, it prevented plasma ghrelin elevation in prolonged fasted states. However, in control rats, food deprivation significantly increased plasma ghrelin levels [24]. This demonstrates the vagus nerve is involved in ghrelin response to changes in energy store. However, vagotomy is not representative of normal physiology. This limits the study’s usefulness in evaluating vagal involvement in ghrelin’s physiological role.

Ghrelin and adiposity

Energy storage forms a vital part of energy homeostasis. Ghrelin induces energy storage in the form of fat by promoting adipose tissue deposition, fatty acid oxidation and reduced energy expenditure [5]. Chronic central ghrelin infusion increased mRNA expression of fat-storage promoting enzymes in white adipocytes of rats. Furthermore, expression of the transcription factors peroxisome proliferator-activated receptor gamma and sterol regulatory element-binding protein 1 was increased. Both transcription factors promote adipogenesis [25]. This seems to support ghrelin’s role in increasing adiposity. Pharmacological ghrelin administration limits the study’s applicability to normal physiology, however.

Both GHSR-KO and ghrelin-KO mice had significantly reduced respiratory quotients upon HFD [9,11]. This shows the absence of ghrelin results in greater utilisation of fat, as opposed to carbohydrates, as an energy substrate. Ghrelin thus appears to promote preservation of fat storage. In line with this, GHSR-KO and ghrelin-KO mice had significantly less white adipose tissue than control mice on HFD [11]. This indicates ghrelin is important in modulating which metabolic substrate is used to maintain energy balance, particularly following HFD exposure. Furthermore, lack of ghrelin function was shown to protect against diet-induced obesity. On a normal diet, only female GHSR-KO mice had significantly less total body adiposity than control mice [11]. There may thus be sexually dimorphic differences in metabolic responses to ghrelin. Ghrelin is potentially more significant in maintaining fat stores in females than males.

Ghrelin also regulates energy homeostasis through brown adipose tissue (BAT). Uncoupling protein-1 (UCP1) in BAT mitochondria is thought to regulate thermogenesis by causing heat dissipation in humans and animals [26,27]. Ageing has been linked to reduced thermogenesis in BAT [28]. Evidence suggests this may be mediated by ghrelin. Elderly GHSR-KO mice displayed increased BAT thermogenesis, with raised UCP1 expression compared to control mice. Significantly higher core body temperature was also detected in elderly GHSR-KO mice [29]. This suggests an increased metabolic rate in GHSR-KO mice, as even a one-degree increase in body temperature could thermodynamically correspond with a ten-percent rise in metabolic rate [30]. Thus, in a similar way to other hormones in animals, ghrelin may play a role in metabolism regulation, resulting in energy expenditure decrease through decreased thermogenesis [31]. However, the mice in this study were of global GHSR-knockout and is thus of limited specificity. Thus it is unknown if the thermogenic effect in BAT was due to a direct action of ghrelin, or through an indirect pathway.

Overall, ghrelin’s effect on fat metabolism and body adiposity levels suggests ghrelin plays an important physiological role in energy storage, and thus energy homeostasis. Ghrelin’s action of increasing adiposity suggests ghrelin inhibition may serve as a potential anti-obesity treatment. Further research is needed to observe if ghrelin exhibits the same effects in humans. Ghrelin’s effect on BAT-regulated thermogenesis also suggests it is important in energy expenditure.

Ghrelin and glucose homeostasis

Evidence points to the major function of ghrelin being control of blood glucose and thus glucose metabolism. Glucose is an important energy source in mammals, thus maintenance of glucose levels is important for energy homeostasis. Ghrelin affects glucose metabolism by influencing both glucagon and insulin levels [2].

Ghrelin secretion from cultured mouse gastric mucosal cells negatively correlated with administered glucose concentration. Insulin administration blocked ghrelin secretion at low glucose concentrations. This suggests ghrelin-releasing cells are directly influenced by glucose concentrations, and low glucose concentrations sensitise ghrelin cells to insulin [32].

Quantitative polymerase chain reaction and histochemistry found GHSR mRNA present in both pancreatic alpha-cells and islets of Langerhans in mice [33]. This suggests the pancreas is a site of direct ghrelin action. Under high glucose conditions, GHSR mRNA was significantly reduced in the two cell types, implying GHSR expression is regulated by ambient glucose levels [31]. Both these studies are limited however, as the use of cells in vitro may not compare with in vivo physiological effect. Furthermore, there may be interspecies variation in pancreatic GHSR expression.

KO models suggest ghrelin’s major physiological role is in glucose metabolism. Ghrelin appears to be crucial in blood glucose control during states of calorie restriction. Both GHSR-KO and ghrelin-KO mice have low blood glucose levels and raised insulin sensitivity under fasted states, but not under fed states [12]. This may be explained by a recent study that identified liver-expressed antimicrobial peptide-2 as an antagonist of GHSR that is released during calorie excess [34,35]. The ghrelin-induced effects on glucose thus appear important during negative energy balance. However, knocking out the ghrelin gene also causes loss of pre-proghrelin, which yields other peptides besides ghrelin [36]. Absence of these in ghrelin-KO mice may influence changes in blood glucose or insulin levels. GOAT-KO mice are unable to acylate ghrelin. Under severe calorie restriction, GOAT-KO mice were unable to control declining blood glucose levels, unlike their control counterparts. After seven days, the mice appeared moribund and were euthanized. Despite this, the mice still exhibited the same food intake and body weight changes as control mice [37]. This shows the importance of acylated ghrelin in maintaining survival through blood glucose control during severe negative energy balance. GHSR antagonists significantly lowered fasting blood glucose levels, again demonstrating ghrelin’s involvement in the homeostatic regulation of blood glucose during fasting [38].

Ghrelin has an inhibitory role on insulin secretion. Ghrelin gene knockout in leptin-deficient mice (a mouse model for obesity) caused a marked reduction in hyperglycaemia. Fasting normalised blood glucose levels. Glucose-stimulated insulin secretion was amplified. C-peptide elevation indicated increased insulin secretion by beta-cells of the pancreas [39]. This is due to ghrelin-KO causing reduced Uncoupling protein-2 (UCP2) mRNA expression in the pancreas. UCP2 is suggested to inhibit insulin secretion [40]. This demonstrates ghrelin’s physiological role in glucose metabolism via insulin level control. Ghrelin also regulates glucose homeostasis through stimulation of glucagon secretion [33]. Re-expression of GHSR in adult GHSR-KO mice caused a rise in glucagon levels and normalisation of blood glucose under caloric restriction [18].

**Figure 2:**
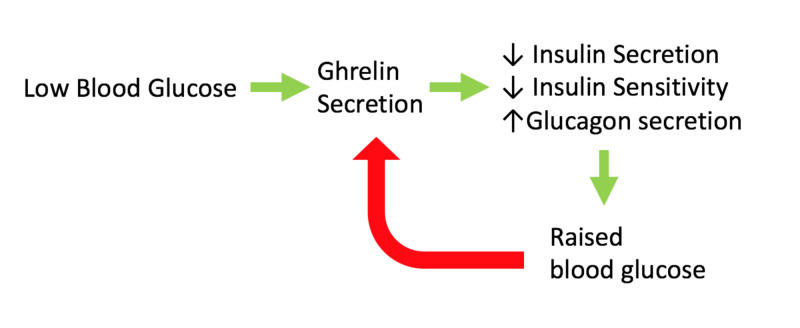
Summary of ghrelin's role in blood glucose control Ghrelin is secreted in response to low blood glucose levels. Ghrelin inhibits insulin secretion and insulin sensitivity, whilst raising glucagon secretion, resulting in a rise in blood glucose. The raised blood glucose then inhibits ghrelin secretion. This demonstrates the essential role ghrelin plays in glucose homeostasis. Red arrow: Inhibition; Green arrow: Stimulation.

Overall, evidence strongly supports ghrelin’s importance in glucose homeostasis. In particular, ghrelin is essential during severe negative energy balance in preventing life-threatening hypoglycaemia. Ghrelin is implied to be important in raising blood glucose (see Figure 2). Evidence suggests ghrelin inhibition may be beneficial in treating type-2 diabetes, in both lowering blood glucose and raising insulin sensitivity. Human studies must be carried out to elucidate ghrelin’s role in glucose homeostasis.

## Conclusions

Ghrelin’s physiological role in energy homeostasis appears to be in modulating energy balance and energy efficiency, mediated through the vagus nerve and hypothalamus. Whilst ghrelin stimulates appetite, it has not been shown that blocking its signalling reduces food intake. Instead, evidence suggests ghrelin’s main role is in adaption of various aspects of energy homeostasis in response to energy imbalance, including an effect of increasing fat storage. Ghrelin also significantly influences glucose homeostasis by decreasing the effect of insulin. Such effects suggest ghrelin inhibition is a potential therapeutic target for type-2 diabetes and obesity.
